# Optimising research for neonates with encephalopathy: the role of core outcome sets

**DOI:** 10.1038/s41390-023-03006-1

**Published:** 2024-01-09

**Authors:** Declan Devane, Shabina Ariff, Malcolm R. Battin, Linda Biesty, Frank H. Bloomfield, Mandy Daly, Patricia Healy, David M. Haas, Jamie J. Kirkham, Vincent Kibet, Sarah Koskei, Shireen Meher, Eleanor J. Molloy, Maira Niaz, Elaine Ní Bhraonáin, Christabell Omukagah Okaronon, Matthew J. Parkes, Farhana Tabassum, Karen Walker, James W. H. Webbe, Fiona A. Quirke

**Affiliations:** 1 Health Research Board – Trials Methodology Research Network (HRB-TMRN), University of Galway, Galway, Ireland; 2 Evidence Synthesis Ireland, University of Galway, Galway, Ireland; 3 Cochrane Ireland, University of Galway, Galway, Ireland; 4 Department of Paediatrics & Child Health, Aga Khan University, Karachi, Pakistan; 5 Department of Newborn Services, Auckland District Health Board, Auckland, New Zealand; 6 Liggins Institute, University of Auckland, Private Bag 92019, Auckland, 1142 New Zealand; 7 Advocacy and Policymaking, Irish Neonatal Health Alliance, Wicklow, Ireland; 8 Department of Obstetrics and Gynecology, Indiana University, Indianapolis, IN USA; 9 Centre for Biostatistics, The University of Manchester, Manchester Academic Health Science Centre, Manchester, UK; 10 Moi University, Cheptiret, Kenya; 11 Institute of Life Course and Medical Sciences, University of Liverpool, Liverpool, UK; 12 Health Research Board Neonatal Encephalopathy PhD Training Network (NEPTuNE), Dublin, Ireland; 13 Department of Paediatrics and Child Health, Trinity College Dublin, Dublin, Ireland; 14 Department of Neonatology, Children’s Hospital Ireland at Crumlin and Tallaght, Dublin, Ireland; 15 Department of Neonatology, Coombe Women and Infants University Hospital, Dublin, Ireland; 16 Family Support Liaison, Irish Neonatal Health Alliance, Wicklow, Ireland; 17 AMPATH, Eldoret, Kenya; 18 NIHR Manchester Biomedical Research Centre, Manchester University NHS Foundation Trust, Manchester Academic Health Science Centre (MAHSC), Manchester, UK; 19 Centre of Excellence in Women and Child Health, Aga Khan University, Karachi, Pakistan; 20 Department of Newborn Care, Royal Prince Alfred Hospital, Sydney, NSW Australia; 21 Faculty of Medicine and Health, University of Sydney, Sydney, NSW Australia; 22 The George Institute for Global Health, Sydney, NSW Australia; 23 Council of International Neonatal Nurses, Sydney, NSW Australia; 24 Academic Neonatal Medicine, Imperial College London, London, UK; 25 School of Medicine, University of Limerick, Limerick, Ireland

We are grateful for the opportunity to reply to the commentary by Gunn et al.,^1^ on our paper ‘COHESION: A Core Outcome Set for the Treatment of Neonatal Encephalopathy.^2^

We agree with their initial assertion that neonatal encephalopathy (NE) remains a significant problem but firmly disagree that the term NE is confusing or misleading. Indeed, the preference for using this term has been the subject of four prior editorials in this journal over 12 years.^3–6^ Common threads in these editorials have been the ‘lack of a consensus’ and concerns about impeding progress. Notably, the three most recent of these^4–6^ recognise the potential for sub-classification of NE by aetiology to influence treatment selection. However, it is also noted that ‘…identifying the precise causal pathway is often challenging’^6^ and the ‘…diagnosis of HIE or asphyxia is often over-utilised in practice and not clinically justified by the limited data at birth.^5^ Given that time is an important factor, with potential interventions needing to be administered early, confirming the aetiology with certainty at the point of trial entry is often impractical. Thus, we consider that using an overarching term, with the potential for subsequent sub-classification based on additional data, is not mutually exclusive.

When developing the core outcome set for NE, we advocated for the term ‘Neonatal Encephalopathy (NE)’ over alternatives, citing its comprehensive nature and encompassing diverse causative factors. Our selection aligns with recommendations from several authoritative bodies, including the American College of Obstetricians and Gynaecologists Task Force on Neonatal Encephalopathy.^7^ We characterise NE as a ‘syndrome of disturbed neurological function in term or late preterm neonates in the first few days of life’, highlighting its broad diagnostic relevance and intentionally avoiding a singular causal focus. Our paper explicitly acknowledges the ambiguity and calls for greater consensus on the terminology, aligning with the editorial perspectives. It draws attention to the inconsistency in how the terms NE and hypoxic-ischemic encephalopathy HIE are used across various clinical studies. This variability was evident in the cooling trials, which used a range of terminologies, including NE, HIE and perinatal asphyxia encephalopathy.^8–12^ Consequently, the term NE was used in the peer-reviewed published protocol for our paper.^13^

The major ongoing challenges in current research include the need for a consensus on the definition of neonatal encephalopathy and the heterogeneity in the outcome measures reported across studies evaluating treatments for neonatal encephalopathy. These issues underscore the necessity for a coordinated, multidisciplinary approach to enhance our understanding and management of this complex condition. Interestingly, two authors of the commentary, Gunn and Ferriero, have recently collaborated with members of our group DEFiNE (Definition of Neonatal Encephalopathy) in a recent editorial which advocates ‘moving from controversy to consensus definitions and subclassification’.^6^ This editorial underscores the challenges arising from the syndromic nature of NE, which can confound families and caregivers. It advocates for approaches to reduce heterogeneity and improve outcomes by identifying the best treatment options. A key initiative is establishing a consensus definition for neonatal encephalopathy, subclassifying subtypes, and stratifying trial entry criteria based on aetiology. Another essential strategy is to standardise outcomes reporting across studies, irrespective of underlying aetiology or type of treatment being investigated. Such standardisation facilitates evidence synthesis through systematic review and meta-analysis, enhancing our understanding of intervention effects^14^ This is the primary aim of our core outcome set.^2^ (ref COHESION paper).^2^

In contrast, the proposal to use the term ‘probable HIE’ may introduce additional uncertainty. This terminology implies a presumed hypoxic-ischemic event as the causative factor for the observed encephalopathy without conclusive evidence. Using ‘probable HIE’ risks confusing the discourse, hinting at a specific aetiology without firm grounding.

We maintain our confidence in the appropriateness of the term ‘NE’. Our paper addresses the need for a better understanding and expression of the complex pathophysiology of neonatal brain injuries. Our Core Outcome Set (COS) is tailored to be broadly applicable across this spectrum, a crucial aspect in scenarios where diagnostic certainty may be elusive. By incorporating outcomes important to healthcare professionals, researchers, and, importantly, parents of affected infants, we ensure that our COS is relevant and responsive to the needs of all key stakeholders in neonatal encephalopathy care and research.
